# Seasonal Changes of Serum Gonadotropins and Testosterone in Men Revealed by a Large Data Set of Real-World Observations Over Nine Years

**DOI:** 10.3389/fendo.2019.00914

**Published:** 2020-01-10

**Authors:** Daniele Santi, Giorgia Spaggiari, Antonio R. M. Granata, Monica Setti, Simonetta Tagliavini, Tommaso Trenti, Manuela Simoni

**Affiliations:** ^1^Unit of Endocrinology, Department of Biomedical, Metabolic and Neural Sciences, University of Modena and Reggio Emilia, Modena, Italy; ^2^Unit of Endocrinology, Department of Medical Specialties, Azienda Ospedaliero-Universitaria of Modena, Ospedale Civile of Baggiovara, Modena, Italy; ^3^Service of Clinical Engineering, Azienda Ospedaliero-Universitaria of Modena, Modena, Italy; ^4^Department of Laboratory Medicine and Anatomy Pathology, Azienda USL of Modena, Modena, Italy

**Keywords:** luteinizing hormone, follicle stimulating hormone, testosterone, seasonal variation, daylight cycle

## Abstract

Environmental rhythmicity is able to affect the hypothalamic-pituitary-gonadal axis in several animals to achieve reproductive advantages. However, conflicting results were obtained when assessing the environmental-dependent rhythmicity on reproductive hormone secretion in humans. This study was designed to evaluate seasonal fluctuations of the main hormones involved in the hypothalamic-pituitary-gonadal axis in men, using a big data approach. An observational, retrospective, big data trial was carried out, including all testosterone, luteinizing hormone (LH) and follicle-stimulating hormone (FSH) measurements performed in a single laboratory between January 2010 and January 2019 using Chemiluminescent Microparticle Immunoassay. Subjects presenting any factor interfering with the hypothalamic-pituitary-gonadal axis were excluded. The trend and seasonal distributions were analyzed using autoregressive integrated moving average (ARIMA) models. A total of 12,033 data, accounting for 7,491 men (mean age 47.46 ± 13.51 years, range 18–91 years) were included. Testosterone serum levels (mean 5.34 ± 2.06 ng/dL, range 1.70–15.80 ng/dL) showed a seasonal distribution with higher levels in summer and a direct correlation to environmental temperatures and daylight duration. LH levels (mean 4.64 ± 2.54 IU/L, range 1.00–15.00 IU/L) presented 2 peaks of secretion in autumn and spring, independently from environmental parameters. FSH levels (mean 5.51 ± 3.24 IU/L) did not show any seasonal distribution. A clear seasonal fluctuation of both LH and testosterone was demonstrated in a large cohort of adult men, although a circannual seasonality of hypothalamic-pituitary-gonadal hormones in humans could be not strictly evolutionarily required. Testosterone seasonality seems independent from LH fluctuations, which could be regulated by cyclic central genes expression, and more sensible to environmental temperatures and daylight duration.

## Introduction

Life is strictly embedded in cyclic changes and several organisms have developed circadian and circannual clocks to adapt their physiological functions to external environmental changes. Accordingly, humans developed circadian clocks to synchronize biological functions to environmental rhythms (1, 2). While abundant evidence is available on daily rhythmicity, less is known about the circannual clock. In particular, the hypothalamic-pituitary-gonadal axis seems to be extremely susceptible to environmental rhythmicity (3), since annual hormone fluctuations are needed for several animals to optimize reproduction timing (4). This physiological mechanism has a genetic substrate, and several clock genes regulate the circadian hormone rhythmicity in a large number of organisms, including mammals (5–9). These genes are expressed in the human hypothalamus, which could be considered the pacemaker of the hypothalamic-pituitary-gonadal axis, and seem to be relevant not only in circadian rhythmicity, but also in seasonal fluctuations. Indeed, several trials confirm the key role of clock genes on both human fertility and testosterone seasonality (10). However, unlike most other animals, humans reproduce throughout the entire year, being able to shield themselves from harsh environmental conditions. Thus, a circannual seasonality of sexual hormones in the human species could not be, evolutionarily, strictly required.

Circannual rhythmicity of several hormones in humans was evaluated so far. Among these, gonadotropins, testosterone and prolactin are the mostly investigated hormones to detect the possible persistence of hormone seasonality. Different times of human life, such as prepuberty or adulthood, were studied to assess the environmental-dependent rhythmicity in hormone secretion (11) with conflicting results. Although hormone efficacy is often dependent on the temporal pattern of secretion (11), as confirmed in several animal models, the role of this ancestral evolutionary mechanism in humans is unclear.

With this in mind, this study was designed to investigate seasonality of reproductive hormones in humans. In particular, we applied a big data approach to highlight the possible circannual secretion of key hormones of the hypothalamic-pituitary-gonadal axis in men in a real-world setting. Indeed, an overall evaluation of the entire endocrine gonadal axis, considering both gonadotropins and testosterone, is needed to comprehensively evaluate whether seasonality still persists in human males. Moreover, this approach could give new light on the hierarchical organization regulating fluctuations in sexual hormone production. Although several papers have been published on this topic, this is the first study based on a big data approach, collecting real-world data and considering a very large dataset collected over a consecutive, 8-year period.

## Materials and Methods

A retrospective observational analysis of a data warehouse was performed on patients living in the Province of Modena, Italy. All laboratory examinations performed from January 2010 to January 2019 at the Department of Clinical Pathology (Ospedale Civile of Baggiovara, Modena, Italy) were included in a large database, enclosing 990,904,591 records. This data warehouse was queried and data of all men older than 18 years who had testosterone, luteinizing hormone (LH) and follicle-stimulating hormone (FSH) measured in the same sample were extracted. All assays were measured on a venous sample taken in the morning after an overnight fast. For each record, the patients' age and the clinical diagnosis were recorded. After data extraction, the clinical data of each patient considered was evaluated according to inclusion and exclusion criteria. Afterwards, serum prolactin (PRL) levels were searched for patients included in the database. Thus, only a subgroup of patients enrolled showed testosterone, gonadotropins and PRL levels.

The resulting dataset was analyzed, calculating the confidence interval at 95% (95% CI) for testosterone, LH and FSH. Only data included in all three 95% CI were included in the final database.

Exclusion criteria were: any kind of hypogonadism, both primary (i.e., Klinefelter syndrome, unilateral and/or bilateral orchiectomy for any reason) and secondary (i.e., Kallmann syndrome, androgen-deprivation therapy for prostate cancer, hyperprolactinemia, complete or partial hypopituitarism). Patients with ongoing androgen replacement therapy were excluded from the dataset. Moreover, if the reason for referral was not available, the corresponding data were excluded from the analysis.

### Hormone Assays

Total testosterone serum levels were measured by Chemiluminescent Microparticle Immunoassay (Architect, Abbott, Dundee, UK), with inter- and intra-assay coefficients of variation (CV) of 5.2 and 5.1%, respectively. FSH and LH were measured by Chemiluminescent Microparticle Immunoassay (Architect, Abbott, Longford, Ireland) with inter- and intra-assay CV of 4.1 and 3.1% for LH, and 4.6 and 4.2% for FSH, respectively. PRL was measured by Chemiluminescent Immunoassay (Beckman Coulter, Brea, CA, USA) with inter- and intra-assay CV of 4.2 and 1.6%, respectively.

The laboratory reference ranges were 2.2–8.7 ng/dL for testosterone, 1–9 IU/L for LH, 1–12 IU/L for FSH and 3–13 ng/mL for PRL. The assay methods and kits used did not change over the years for all hormones considered.

### Semen Analysis

The data warehouse was queried to extract available semen analyses of patients with a complete hormonal evaluation of the pituitary-gonadal axis. Semen analyses were performed following the most recent World Health Organization (WHO) guideline (12). The following seminal parameters were included: volume (mL), total sperm number (millions), sperm concentration (millions/mL), percentage of normal/abnormal forms (%), percentage of motile sperms (%) and pH.

### Seasonal Assessment

The effect of seasonal changes was considered connecting hormonal data to humidity and maximum, minimum, and mean daily temperatures registered on the day of blood sample collection. Temperature data were obtained using a meteorological model, CALMET, developed at the Hydro Meteorological Service of the Emilia-Romagna environmental protection agency (ARPA) (https://www.arpae.it). Sites of evaluation of environmental temperatures were used to localize the recording unit and connecting it to the residential address of each patient. In order to consider the diurnal rhythm, the number of daylight hours was calculated using the Sunrise-Sunset Calendar of SunEarthTools (https://sunrise-sunset.org/api).

### Statistical Analysis

Data distribution was evaluated performing Kolmogorov-Smirnov test. Correlations among data were performed by Pearson or Spearman tests, for normal and not-normal distributed parameters, respectively. Considering that usual statistical analyses could be biased in large dataset, resampling methods were applied to confirm the regression analyses. To this purpose, the k-fold cross-validation method was selected (13, 14). We randomly split all data into 5 folds, then we used 4 folds for training and 1 fold for testing the result. This internal 5-fold cross-validation test was repeated 100 times. The average regressions obtained by each model were finally compared to the usual statistical approach.

Testosterone, LH and FSH distribution was evaluated considering the date of examination by autocorrelation analyses. Autocorrelation functions were first calculated as lag 1, which is the correlation between adjacent observations in a time series (15, 16). Autocorrelation function represents the statistical approach to measure the linear relationship between an observation at specific time and the observations at previous times. Since our dataset included a large number of data, autocorrelation functions were repeated increasing lag number, from 1 (default) to 100. Subsequently, the partial autocorrelation functions were calculated by the correlation of the transformed time series, aiming at identifying the order of an autoregressive model. The Box-Ljung test was used at inspecting the autocorrelations among residuals and to determine the seasonal model (17).

When the autocorrelation functions suggested a seasonality, seasonal decomposition was applied. The Wilcoxon Signed Rank test was used to detect seasonality (18). Once a seasonality was suggested, the autoregressive integrated moving average (ARIMA) model was used to quantify the seasonality pattern detected. ARIMA is a generalization of an autoregressive moving average (ARMA) model, created to better understand the series data distribution. ARIMA models were defined by three letters (p,d,q), where parameters p, d, and q are non-negative integers, p is the order (number of time lags) of the autoregressive model, d is the degree of differencing (the number of times the data have had past values subtracted), and q is the order of the moving-average model. The auto-ARIMA function was used to select the best model to be applied to describe time series distribution. Since the ARIMA models could have drawbacks when applied to large dataset (with more than 200 data), we re-tested the ARIMA model considering the last year of observation, reducing the sample size. The Holt Winters method was used to detect alpha coefficient for correction of distribution. The prediction of seasonality was considered for alpha coefficient between 0.01 and 0.30. Finally, the Ljung–Box test (*h* = 50) was used to detect seasonality, considering whether any of a group of autocorrelations of a time series was different from zero (19–21).

In order to investigate whether seasonal peaks were detectable, the entire original dataset was divided in four groups according to season in which the blood samples were taken: winter, spring, summer and autumn. The four seasons were recognized using the following solstices and equinoxes dates: 21st June, 22th December, 20th March, and 23th September. The mean values of testosterone, LH and FSH were compared among seasons by Kruskal-Wallis test. *Post hoc* analyses were performed by Tukey test. Moreover, in order to evaluate the role of age on sexual hormone variations, the entire cohort was divided in quartiles according to age distribution. Thus, testosterone, LH and FSH distribution among seasons was evaluated in each quartile of patient's age. In order to evaluate whether seasonal distribution was maintained considering only hormonal values within the laboratory reference ranges, patients were divided in 3 subgroups: (i) below, (ii) within, and (iii) above the laboratory reference ranges. These ranges are used to excluding outliers from the analysis.

In order to evaluate the role of environment on sexual hormones, bivariate correlations were performed among testosterone, LH and FSH from one side and maximum, minimum and mean temperatures, humidity and daylight duration using Rho's Spearman correlation. In this setting, in the subgroup of patients with available PRL measurements, the seasonal changes of PRL were evaluated. Thus, testosterone and gonadotropins were correlated to PRL serum levels using Rho's Spearman correlation.

Statistical analysis was performed using the “Statistical Package for the Social Sciences” software (version 25.0; SPSS Inc., Chicago, IL) [Research Resource Identifier (RRID):SCR_002865] and RStudio Server Open Source Edit Version 0.99.902 2016 and R programming software (RRID:SCR_000432). For all comparisons, *p*-values < 0.05 were considered statistically significant.

### Ethical Statement

All procedures performed were in accordance with the ethical standards of the Helsinki Declaration of 1975 as revised in 2013. Considering the retrospective study design, it was not possible to obtain informed consent from all participants included in the study, but all examinations were approved by the Hospital management, since data were collected anonymously.

## Results

From the 17,650 rows first extracted, 14,131 data remained after inclusion and exclusion criteria evaluation. 12,033 data were included in the final overall database, accounting for 7,491 men (mean age 47.46 ± 13.51 years, min 18, max 91 years). Mean testosterone serum levels ranged from 1.70 to 15.80 ng/dL (mean 5.34 ± 2.06 ng/dL), LH ranged from 1.00 to 15.00 IU/L (mean 4.64 ± 2.54 IU/L) and FSH from 0.40 to 16.30 IU/L (mean 5.51 ± 3.24 IU/L). The three parameters were not normally distributed (*p* < 0.001).

Semen analyses were available only in 2.6% of the cohort (317 patients), with a mean sperm concentration of 55.83 ± 26.48 millions/mL, progressive motility 35.75 ± 22.44%, non-progressive motility 9.22 ± 8.11%, typical forms 3.99 ± 3.09% and a mean volume of 3.64 ± 1.93 mL. PRL serum levels were available in 31.8% of the cohort (3,830 patients) with a mean of 11.50 ± 6.36 ng/mL (minimum 0.30 and maximum 62.20 ng/mL).

### Seasonal Decomposition

**Figure 1:**
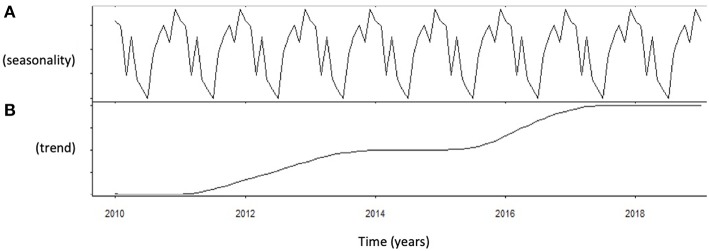
Analysis of serum testosterone level distribution using autoregressive integrated moving average (ARIMA) model. **(A)** Shows the data distribution across years, suggesting possible peak and nadir. **(B)** Shows the trend of data collected across years of observation.

**Figure 2:**
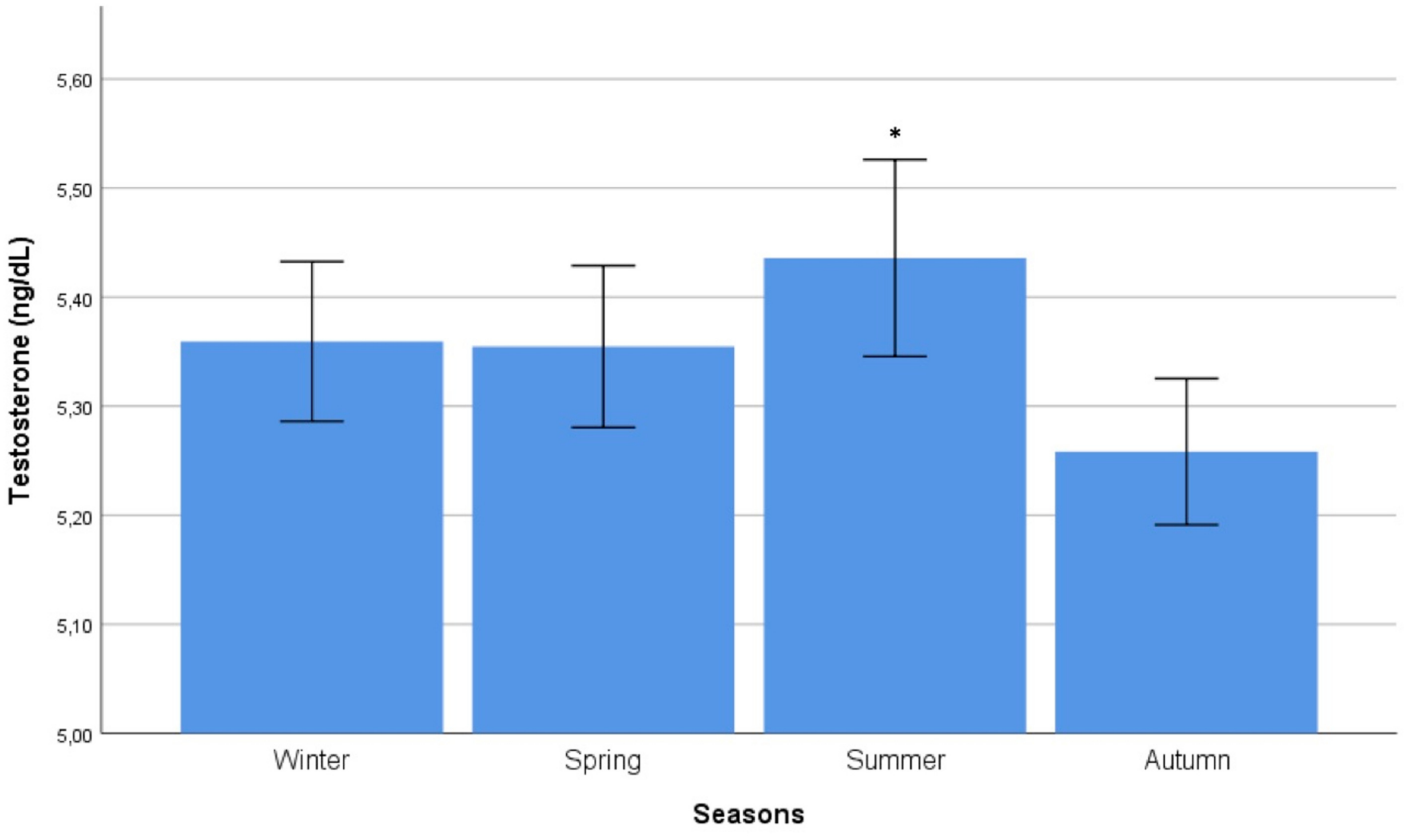
Comparison of mean testosterone serum levels among seasons. Comparison was performed by Kruskal-Wallis test. ^*^Identifies the highest testosterone serum levels at *post hoc* Tukey test.

Autocorrelation function was applied to testosterone distribution, identifying two significant peaks followed by a long exponential tail, typical of historical series (peak 1: 0.178, standard error 0.009, coefficient 380.13, Box-Ljung test, *p* < 0.001; peak 2: 0.045, standard error 0.009, coefficient 490.64, Box-Ljung test, *p* < 0.001). This double peak suggests the existence of a seasonal component in an annual period. Hypothesizing a monthly change, seasonal decomposition was applied, setting the correction factors for seasonality. The Wilcoxon Signed Rank test confirmed the seasonal distribution (*p* = 0.001). ARIMA models were applied to evaluate quantitatively the seasonal testosterone pattern. The auto-ARIMA test selected the ARIMA (2,0,9) as the best applicable model, with mean 4.59 and standard error 0.50, depicting following coefficients: sigma^2^ estimated 2.78 with log likelihood = −18.78, Akaike's information criterion (AIC) = 41.55 and Bayesian information criterion (BIC) = 42.16 (Figure 1). This result was confirmed considering only the last year of observation (sigma^2^ estimated 2.82 with log likelihood = −17.02). From the distribution analysis, testosterone showed a significant trend across the years (Figure 1B), together with a seasonal distribution (Figure 1A), confirmed at the Box-Ljung test (X-squared = 10.989, degrees of freedom = 8, *p*-value = 0.022). The d = 0 parameter represents the stationary time series, which was not confirmed by our results (Figure 1B). Thus, we run the ARIMA (1,1,1) model in which d = 1 represents a stochastic trending component, confirming the seasonality previously reported (mean 1.84 and standard error 0.10, sigma^2^ estimated 1.84 with log likelihood = 38.4, AIC = 61.39 and BIC = 75.18). The analysis of testosterone difference among seasons was performed to detect the zenith. Testosterone serum levels were significantly different among seasons (*p* = 0.013), with higher levels in summer compared to autumn (*p* = 0.008) (Table 1, Figure 2).

**Table 1:** Hormone serum levels in the four seasons across the study years.

		**Number of samples**	**Mean**	**Standard deviation**
Testosterone (ng/dL)	Winter	3,199	5.36	2.07
	Spring	3,140	5.35	2.08
	Summer	2,181	5.44	2.11
	Autumn	3,513	5.26	1.99
	*p*-value	**0.013**		
LH (IU/L)	Winter	3,199	4.56	2.53
	Spring	3,140	4.78	2.63
	Summer	2,181	4.44	2.34
	Autumn	3,513	4.72	2.59
	*p*-value	**<0.001**		
FSH (IU/L)	Winter	3,199	5.52	3.23
	Spring	3,140	5.72	3.36
	Summer	2,181	5.31	3.16
	Autumn	3,513	5.44	3.19
	*p*-value	0.202		

*P-values are obtained by Kruskall–Wallis test*.

*FSH, follicle-stimulating hormone; LH, luteinizing hormone*.

*Bold values represent statistical significant results*.

**Figure 3:**
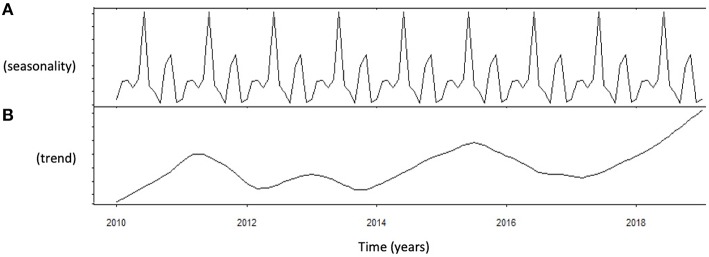
Analysis of luteinizing hormone (LH) levels distribution using autoregressive integrated moving average (ARIMA) model. **(A)** Shows the data distribution across years, suggesting possible peak and nadir. **(B)** Shows the trend of data collected across years of observation.

**Figure 4:**
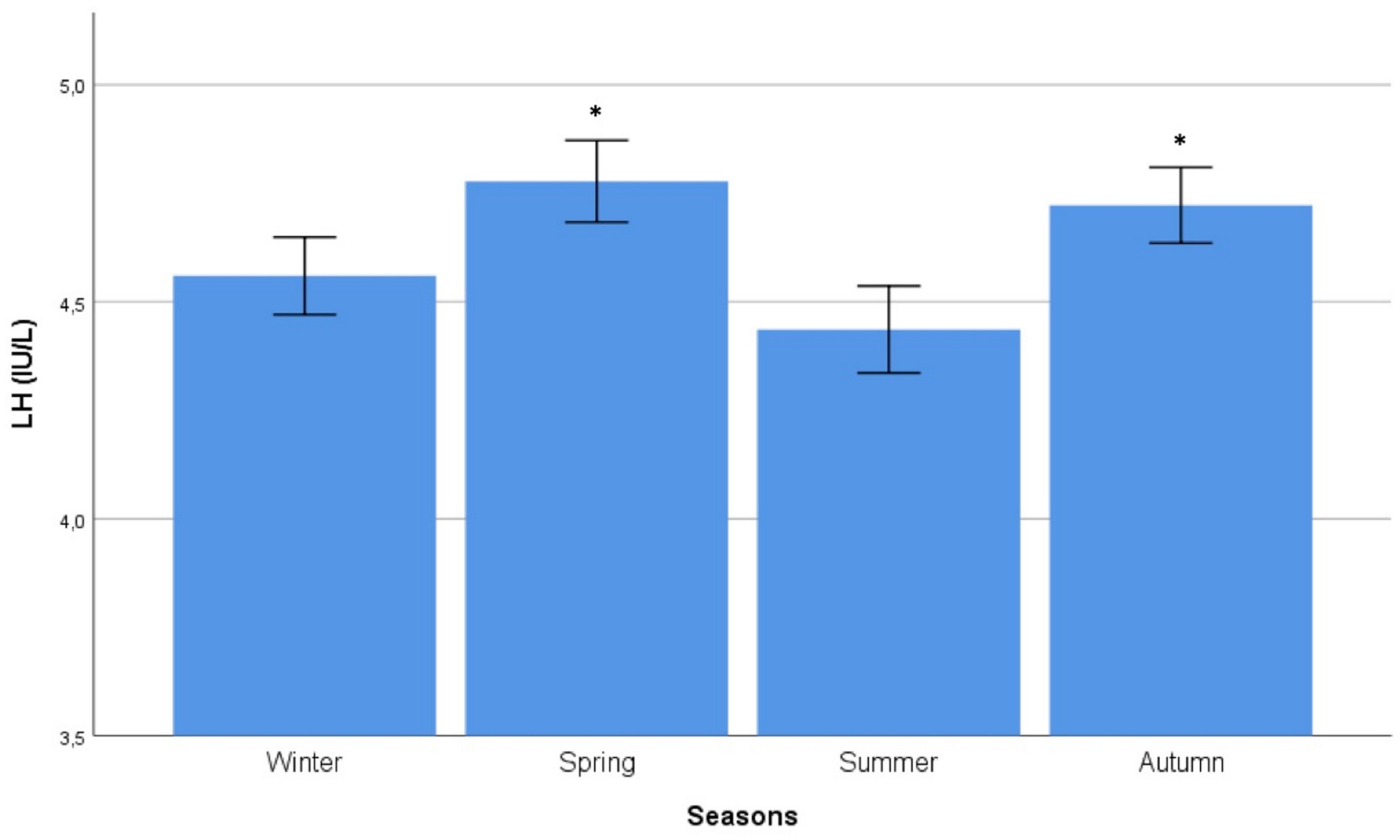
Comparison of mean luteinizing hormone (LH) serum levels among seasons. Comparison was performed by Kruskal–Wallis test. ^*^Identifies the highest LH serum levels at *post hoc* Tukey test.

Autocorrelation function was applied to LH distribution, identifying two significant peaks followed by a long exponential tail, typical of historical series (peak 1: 0.216, standard error 0.009, coefficient 562.7, Box-Ljung test, *p* < 0.001; peak 2: 0.108, standard error 0.009, coefficient 1928.0, Box-Ljung test, *p* < 0.001). This double peak was confirmed using the Wilcoxon Signed Rank test (*p* = 0.001), confirming the seasonal LH distribution. Seasonal decomposition was applied with ARIMA (0,0,0), detecting mean value 3.91 and standard error 0.54, sigma^2^ estimated 3.29 with log likelihood = −19.62, AIC = 43.23, and BIC = 43.84. LH did not show any significant trend across the years (Figure 3B). This result was confirmed considering only the last year of observation (sigma^2^ estimated 3.11 with log likelihood = −18.30). A seasonal distribution was evident (Figure 3A), with two annual peaks. Mean LH levels confirmed a different distribution among the seasons (*p* < 0.001), with two peaks per year, in spring and autumn, respectively (Figure 4, Table 1). Indeed, LH serum levels were significantly higher in spring compared to summer and winter (*p* = 0.004 and *p* < 0.001, respectively) and in autumn compared to winter and summer (*p* = 0.044 and *p* < 0.001, respectively) (Figure 4, Table 1).

Considering FSH, no significant peaks were detected by autocorrelation functions. As a confirmation, ARIMA did not highlight any seasonal distribution of FSH (mean 3.82, standard error 0.44, sigma2 estimated 2.14, log likelihood = −17.47, AIC = 38.93 and BIC = 39.54), and the Box-Ljung test did not detect any significant seasonal distribution (X-squared = 9.44, degrees of freedom = 8, *p* = 0.306). Mean FSH levels differences among seasons confirmed the lack of seasonality (*p* = 0.202) (Table 1).

Considering patients' age, the following quartiles were created: (i) from 18 to 35 years (group 1 – 3145 data), (ii) from 35.1 to 48 years (group 2 2880 data), (iii) from 48.1 to 57 years (group 3 – 3310 data), and (iv) older than 57.1 years (group 4 – 2698 data). In the first group, LH and testosterone did not differ among seasons (*p* = 0.773 and *p* = 0.301, respectively). In group 2, LH was significantly different among seasons (*p* < 0.001), confirming the highest levels in spring and autumn (*p* < 0.001 and *p* = 0.005, respectively). However, annual peak of testosterone was not confirmed (*p* = 0.060). In group 3, the seasonal differences of both testosterone and LH were confirmed (*p* = 0.004 and *p* = 0.002, respectively). At *post hoc* analysis, the highest testosterone levels were detected in summer (*p* = 0.002) and the highest LH levels in spring and autumn (*p* = 0.004 and *p* = 0.006, respectively). Finally, in group 4, no seasonal differences were detected, neither for testosterone nor for LH (*p* = 0.155 and *p* = 0.080, respectively).

Considering semen analyses, sperm concentration was used to evaluate seasonality. Autocorrelation function did not detect significant peaks and no seasonality was detected at Box-Ljung test (*p* = 0.402). Finally, PRL did not show any seasonal fluctuation (*p* = 0.421), without significant differences among seasons (*p* = 0.181).

### Correlations Among Hormones

Patients' age was inversely related to serum testosterone levels (*R* = −0–148, *p* < 0.001) and directly related to LH (*R* = 0.185, *p* < 0.001) and FSH (*R* = 0.281, *p* < 0.001). As expected, total testosterone serum levels were directly related to LH (*R* = 0.147, *p* < 0.001), but not to FSH (*R* = −0.006, *p* = 0.482). Finally, LH was directly related to FSH (*R* = 0.538, *p* < 0.001). The internal 5-fold cross-validation method confirmed the significant result obtained by conventional statistical analyses. No correlations among semen parameters and sexual hormones were detected.

### Environmental Influence on Seasonality

Testosterone serum levels were within the laboratory reference range (2.2–8.7 ng/dL) in 10,905 patients (90.6%), while 311 patients (2.6%) and 817 patients (6.8%) showed testosterone levels lower and higher than the reference range. Although testosterone seasonality remained statistically significant considering only data within the reference range, a significant zenith was not detected by mean differences among seasons (*p* = 0.288) (Table 2), suggesting that the significant seasonal variability is evident including values that are outside the laboratory reference range. On the contrary, LH seasonality was confirmed for data within the reference range (1–9 IU/L) and higher levels were confirmed in spring and autumn (Table 2) (*p* = 0.001).

**Table 2:** Hormone serum levels in the four seasons across the study years considering only hormones within the laboratory reference ranges.

		**Number of samples**	**Mean**	**Standard deviation**
Testosterone (ng/dL)	Winter	2,891	5.11	1.62
	Spring	2,827	5.14	1.68
	Summer	1,958	5.13	1.61
	Autumn	3,229	5.08	1.64
	*p*-value	0.288		
LH (IU/L)	Winter	2,949	4.30	2.25
	Spring	2,878	4.46	2.32
	Summer	2,059	4.22	2.05
	Autumn	3,222	4.45	2.32
	*p*-value	**0.001**		
FSH (IU/L)	Winter	2,985	5.21	2.81
	Spring	2,895	5.38	2.97
	Summer	2,049	5.02	2.70
	Autumn	3,306	5.19	2.89
	*p*-value	0.312		

*P-values are obtained by Kruskall–Wallis test*.

*FSH, follicle-stimulating hormone; LH, luteinizing hormone*.

*Bold values represent statistical significant results*.

**Figure 5:**
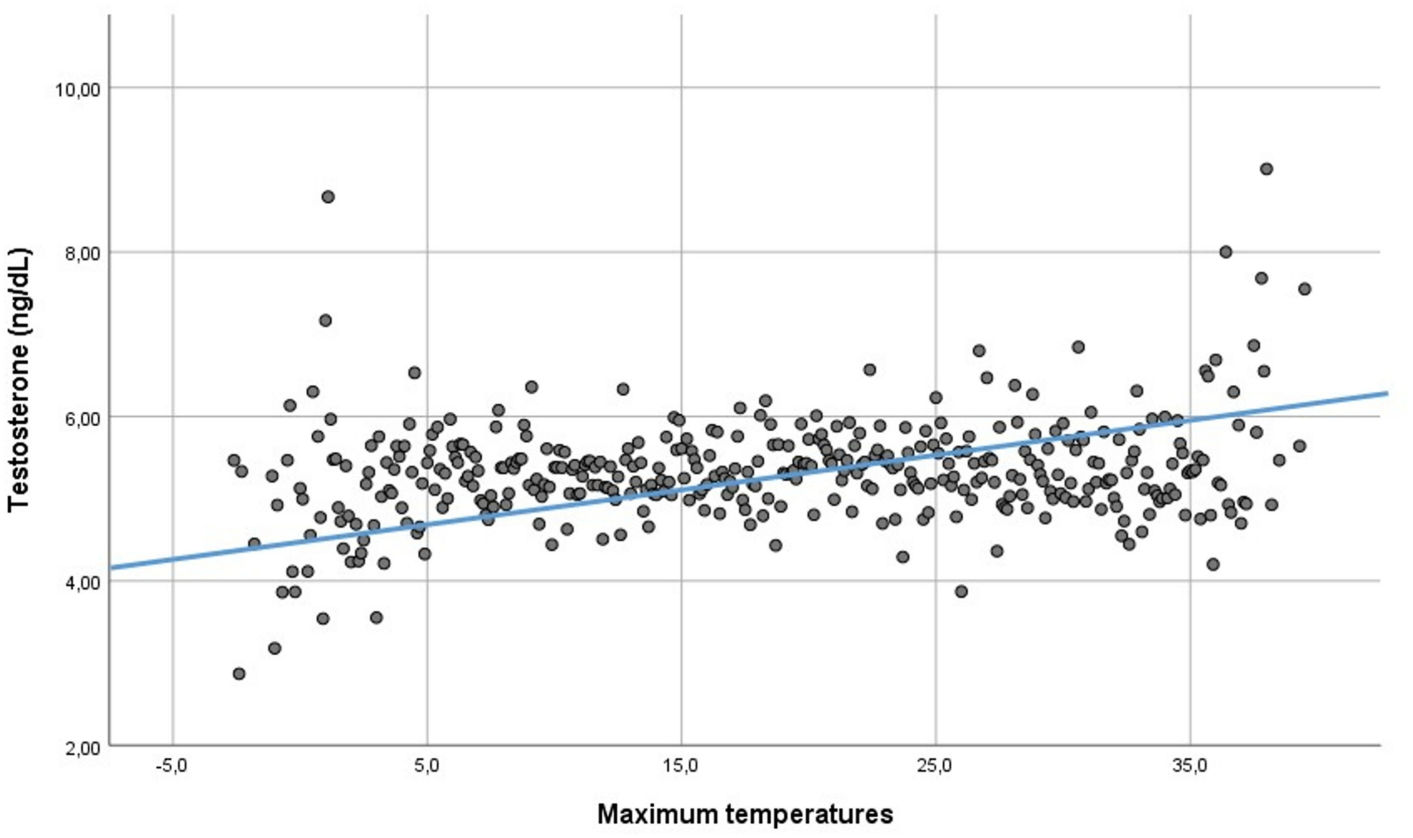
Linear regression between testosterone serum levels and environmental maximum temperatures.

PRL serum levels did neither correlate with testosterone (Rho: 0.002, *p* = 0.804), nor LH (Rho: 0.005, *p* = 0.665) nor FSH (Rho: 0.006, *p* = 0.734). Serum total testosterone was directly related to maximum, minimum and mean daily temperatures (Rho: 0.019—*p* = 0.041, Rho: 0.023—*p* = 0.011, and Rho: 0.021—*p* = 0.024, respectively) (Figure 5), but not to humidity (Rho: −0.009, p = 0.340). Moreover, testosterone was directly related to daylight duration (Rho: 0.021—*p* = 0.020). LH was directly related to minimum temperatures (Rho: −0.022—*p* = 0.018), but not to maximum and mean temperatures, humidity and daylight duration (Rho: 0.012—*p* = 0.173, Rho: 0.016—*p* = 0.089, Rho: 0.012—*p* = 0.202, and Rho: 0.007 – *p* = 0–467, respectively). The cross-validation method confirmed the significant correlations, apart from the correlation between LH and minimum temperatures. Indeed, after cross-validation, this correlation was lacking, suggesting that the large amount of data biased this correlation. FSH was not related to environmental parameters. Finally, after seasonal decomposition, environmental temperatures (maximum, minimum and mean temperatures) showed a significant increasing trend across years (*p* < 0.001). In particular, the mean yearly temperature passed from 13.13 ± 8.28 °C in 2010 to 14.61 ± 7.81 °C in 2018. Thus, the increasing trend detected in testosterone distribution could be related to the increasing environmental temperature.

## Discussion

We demonstrate a clear seasonal fluctuation of both LH and testosterone in a large sample of adult human males. As expected, testosterone appears directly related to LH, but the annual fluctuation of these two hormones is not synchronous. LH shows a bi-annual fluctuation, with two peaks reached in spring and autumn, while testosterone shows only one summer peak. Moreover, the testosterone annual change shows a wider variability in annual values compared to LH, evident including levels below and above the laboratory reference ranges, while LH fluctuations remain irrespective of the reference range. Interestingly, the testosterone zenith is reached at least 3 months after the LH peak, a possible late consequence of the vernal LH peak. However, should this rhythmicity reflect a connection between the pituitary gland and the testicle, we should find two testosterone peaks every year. Rather, testosterone seasonal fluctuation could be mainly influenced by the environment. In particular, we show here an increasing trend of environmental temperatures across the years of observation, related to increasing testosterone serum levels. Moreover, when temperatures are higher during the year and the daylight duration is the longest (i.e., summer), testosterone serum levels reach their annual zenith. On the contrary, LH seasonality seems to be independent from environment, and a central mechanism, possibly regulating seasonal fluctuations of the hypothalamic gonadotropin-releasing hormone (GnRH), might be involved. It is well-known that LH secretion is the result of GnRH pulsatility, regulated by hypothalamic clock genes from one side (22) and pulsatile secretion of Kisspeptin on the other side (23), which is the main regulatory mechanism of GnRH secretion in vertebrates (24, 25). In seasonally breeding animals, the circadian and photoperiodic regulation of the neuroendocrine system is largely demonstrated to modulate diurnal and semilunar spawning rhythm (23). Similarly, a complex regulation of hormonal seasonality, involving the pineal pulse generator, is suggested in humans (26). In particular, the melatonin secretory cyclic pattern seems to be sufficient to compensate the physiological secretory pattern which is lacking in men with congenital GnRH deficiency (26). Our study does not show any influence of daylight duration on LH secretion, suggesting a mechanism probably independent from melatonin.

In the literature, 15 clinical trials investigated testosterone seasonality (Table 3). Ten studies detected testosterone fluctuations along the year (66.7%) (28–30, 35–41), with an annual pattern of testosterone secretion highlighted in most cases, and a bi-annual pattern detected only in 3 out of 10 studies (Table 3), whereas 5 did not (27, 31–34). Almost all previous studies evaluated small groups of men and only 3 studies considered more than one thousand subjects. Moreover, two of the most numerous casuistries enrolled older (32) and younger (30) men separately, whereas in our study we cover the entire life-time after puberty, from 18 to 91 years. Only Svartberg et al. evaluated a large cohort including men of all ages older than 25 years (41). However, the seasonal evaluation was limited to testosterone serum levels. A comprehensive assessment of the seasonal rhythmicity of the pituitary-gonadal axis requires not only a high number of adult patients without age limits but also all hormones involved. In our study we could evaluate how the seasonal hormonal changes were affected by age. Indeed, dividing the entire cohort of patients according to age, we highlight seasonal changes in men between 35 and 57 years, whereas no seasonal effect seems evident for men younger than 35 years or older than 57 years. This finding is novel and could explain the discrepancies of the results reported in the earlier literature. Hormones seasonality is lost after 57 years, when a progressive decline of testosterone occurs, probably limiting the yearly change (42).

**Table 3:** Published trials on testosterone seasonality.

	**Number of men**	**Age of men evaluated (years)**	**Patients' characteristics**	**Country**	**Testosterone seasonality**	**LH seasonality**	**FSH seasonality**
Abbaticchio et al. (27)	248	Mean ± SD: 28.9 ± 7.5	Infertile men	Italy	Not detected	Not detected	Not detected
Bellastella et al. (28)	106	Range: 6–10	Pre-pubertal	Italy	AP: summer	AP: winter	Not detected
Bellastella et al. (29)	10	Range: 25–30	Healthy men	Italy	AP: autumn	AP: spring	Not detected
Dabbs et al. (30)	4,462	Range: 32–44	Military veterans	United States	AP: autumn	Not evaluated	Not evaluated
Dai et al. (31)	243	Range: 35–73	Multiple Risk Factor Intervention Trial	United States	Not detected	Not evaluated	Not evaluated
Lee et al. (32)	3,369	Range: 40–79	European Male Aging Study	Europe	Not detected	Not evaluated	Not evaluated
Maes et al. (33)	13	Mean ± SD: 38.7 ± 13.4	Healthy men	Belgium	Not detected	Not evaluated	Not evaluated
Martikainen et al. (34)	22	Not available	Young men	Finland	Not detected	Not detected	Not detected
Meriggiola et al. (35)	16	Not available	Healthy men	Italy	AP: summer	AP: summer	AP: summer
Nicolau et al. (36)	63	Mean ± SD: 77.0 ± 8.0	Healthy men	Romania	Annual	Annual	Not detected
Perry et al. (37)	65	Range: 70–102	African-American males	United States	AP: winter	Not evaluated	Not evaluated
Reinberg et al. (38)	260	Median: 32	Men undergoing vasectomy	France	AP: autumn	AP: autumn	AP: summer
Sawhney et al. (39)	9	Not available	Healthy men	Antarctica	Bi-AP: summer and autumn	Not detected	Not detected
Smals et al. (40)	15	Mean ± SD: 33.5 ± 5.9	Healthy men	United States	Bi-AP: summer and autumn	Not evaluated	Not evaluated
Svartberg et al. (41)	1548	Older than 25	Healthy men	Norway	Bi-AP: winter and autumn	Not evaluated	Not evaluated

*AP, annual peak; FSH, follicle-stimulating hormone; LH, luteinizing hormone; SD, standard deviation*.

The seasonal variability could be due to environmental influence on the reproductive system (4). Available studies evaluating testosterone fluctuations are not homogeneously distributed across the world and only few latitudes have been studied so far. In this context, the lack of sun exposure for a long period of the year, as observed at high latitude countries (34, 39, 41), could represent a confounding factor in evaluating the hormonal seasonality. Indeed, daily hours of sunshine, minimum and maximum temperatures and humidity were demonstrated to influence annual rhythms of human reproduction already in the 1930s (4) and a relationship between testosterone and melatonin secretion has been suggested (43). However, after industrialization, humans are progressively and increasingly shielded from both daylight duration by indoor work, and environmental temperature by heating and air conditioning. These changes in life habits might result in a “de-seasonalization” of human reproduction and possibly in testosterone fluctuation. However, as shown in the majority of industrialized populations studied so far, we confirm the persistence of an annual pattern of testosterone fluctuation. Moreover, we confirm the correlation between testosterone and environmental temperatures, considering maximum, minimum and mean daily values. Increasing environmental temperatures, testosterone raises, reaching the highest values in summer. In this setting, there is large evidence of the detrimental effect of local heat on Leydig cells activity and survival in animal models (44, 45). In particular, heat-induced testicular damage is mediated by the activation of specific apoptotic pathways in animal models (46, 47). However, less is known about the possible effect of environmental temperature on Leydig cells activity in humans. Here we detect a direct linear correlation between testosterone and environmental temperatures, suggesting that low environmental temperatures may be less favorable for testicular steroidogenesis.

Apart from the seasonal fluctuation, testosterone showed a significant increasing trend during years, from 2010 to 2018. This trend could be explained by the increasing environmental temperatures, recorded in the years of the study. Indeed, we demonstrated a direct relationship between testosterone and environmental temperatures in our cohort. Accordingly, environmental temperatures increased in the 9-years interval of the study, with a mean increase of 1.48°C. This increase goes along with a mean testosterone increase of 0.44 ng/dL detected after 9 years of evaluation.

In our cohort, FSH does not fluctuate and a seasonal change in sperm parameter is not detected. However, semen analyses were available only in 2.6% of the entire group, limiting the statistical power. Indeed, increasing the sample size (5,573 semen analyses), we previously detected semen seasonality, with higher sperm number in winter/spring seasons compared to summer/autumn (48). Moreover, in this previous work, a significant correlation between semen analyses and environmental parameters was evident (48). Thus, a larger dataset, containing both semen analyses and hormone evaluations, is needed to completely understand the environmental influence on reproduction along the seasons.

Our study has some strengths. We evaluated (i) a large number of men, (ii) living at the same latitude, (iii) in a long time-frame interval, (iv) without known diseases affecting the hypothalamic-pituitary-gonadal axis, and (v) considering both testosterone and gonadotropins serum levels. However, several limitations should be considered. First, patients were evaluated only once, thus a longitudinal evaluation of testosterone changes in the same patient is not possible. Second, testosterone serum levels were assayed using commercially available kits and not the gold-standard liquid-chromatography tandem mass-spectrometry (LC-MS/MS). Third, no information is available about liver function. Thus, we are not able to consider possible sex hormone binding globulin (SHBG) changes and, consequently, whether these fluctuations are reflected in a seasonal variation of bioavailable testosterone. Finally, semen analyses were available only in a small subgroup of patients and an overall assessment of the seasonality of FSH and semen parameters together is prevented. An accurate evaluation of the seasonal influence on spermatogenesis could elucidate the possible residual role of environmental exposure in terms of reproductive advantage. In this context, the coeval fluctuation of androgens could be involved, maybe influencing libido to optimize conceptions.

## Conclusions

In conclusion, our results demonstrate biannual/circannual fluctuations of serum LH and testosterone, suggesting a seasonal influence on the pituitary-gonadal axis in the human species. The circannual testosterone and LH fluctuation is possibly subjected to different regulation mechanisms (central for LH vs. environmental for testosterone). Considering the limited amplitude of the testosterone and LH fluctuation across the year, the absence of seasonality in the youngest and oldest age groups, and the reduced exposure to environmental factors in the industrialized era, we could speculate that the ancestral secretory pattern adaptive toward seasons in various animal species is (gradually?) disappearing in the human.

## Data Availability Statement

The data that support the findings of this study are available on request from the corresponding author. The data are not publicly available due to privacy or ethical restrictions.

## Ethics Statement

Ethical approval was not provided for this study on human participants because considering the retrospective, big data study design, it was not possible to obtain informed consent from all participants included in the study, but all examinations were approved by the Ospedale Civile of Baggiovara management, since data were collected anonymously. Written informed consent for participation was not required for this study in accordance with the national legislation and the institutional requirements.

## Author Contributions

DS conceived the study, analyzed the data, and wrote the manuscript. GS analyzed the data and wrote the manuscript. MSe coordinated the data extraction. ST and TT performed laboratory assays. AG and MSi contributed wrote the manuscript. All authors edited the manuscript or revised it critically for important intellectual content and approved the final draft.

### Conflict of Interest

The authors declare that the research was conducted in the absence of any commercial or financial relationships that could be construed as a potential conflict of interest.
